# MARITAL SEQUENCING ACROSS THE LIFE COURSE: RACE AND COHORT INTERSECTIONS

**DOI:** 10.1093/geroni/igac059.547

**Published:** 2022-12-20

**Authors:** James Iveniuk, Riddhi Gupta

**Affiliations:** NORC at the University of Chicago, Chicago, Illinois, United States; NORC at the University of Chicago, Chicago, Illinois, United States

## Abstract

In this paper, we undertake an analysis of marital/cohabitation sequences over a person’s entire life course, examining intersections between race and cohort. We draw upon data from a nationally representative survey of older Americans (collected in 2005/2006; N=3005). Using optimal matching and cluster analysis, we find three clusters: those who have been without a partner for many years (10%), those who recently lost a spouse (27%), and those who are still married (62%). All three clusters tended to marry young; only 2% never married or cohabited. Non-Hispanic Black respondents were far less likely than Non-Hispanic White respondents to be in the still-married cluster. The oldest Hispanic respondents were also more likely to be in the recently-unpartnered cluster, compared to younger Hispanic respondents. Cluster membership was also associated with being married ten years later (2015/2016; 1535 retained), with the longstanding-unpartnered cluster less likely to be married, compared to the recently-unpartnered cluster.

